# The Current Status in Terms of Vaccination for Individuals Infected with Human Immunodeficiency Virus

**DOI:** 10.3390/v17020171

**Published:** 2025-01-25

**Authors:** Nan Xu, Yanru Shen, Weijin Huang, Jianhui Nie

**Affiliations:** 1 Division of HIV/AIDS and Sex-Transmitted Virus Vaccines, National Institute for Food and Drug Control (NIFDC), State Key Laboratory of Drug Regulatory Science, Beijing 102629, China; xunan@nifdc.org.cn (N.X.); yanru0613@sina.com (Y.S.); 2 Division of HIV/AIDS and Sex-Transmitted Virus Vaccines, National Institute for Food and Drug Control (NIFDC), NHC Key Laboratory of Research on Quality and Biological Products, Beijing 102629, China

**Keywords:** HIV, HIV-infected individual, vaccination, safety, effectiveness

## Abstract

Human immunodeficiency virus (HIV)-infected individuals have an increased risk of various infections due to their impaired host immune system, resulting in higher morbidity and mortality rates. These patients severely suffered during the COVID-19 epidemic, the influenza epidemic and the spread of monkeypox. Reducing serious infections is one of the most important measures to improve HIV-infected individuals’ quality and length of life. Based on the preparation processes and their antigenic properties, vaccines are divided into several types, including inactivated vaccines, attenuated live vaccines, recombinant protein vaccines, toxoid vaccines, polysaccharide vaccines, polysaccharide (protein) combined vaccines, nucleic acid vaccines, viral vector vaccines, etc. With the innovation of vaccine preparation technology in recent years and the acceleration of vaccine approval and market launch, more and more vaccine products suitable for HIV-infected individuals have become available. Because of their deficient immune systems, the type of vaccines and the schedule of vaccinations available to individuals living with HIV are sometimes different from those with healthy immune systems. This article reviewed the current status of vaccination in and shed light on the vaccination strategies for HIV-infected persons in terms of their safety and effectiveness.

## 1. Overview

Human immunodeficiency virus (HIV) belongs to the retrovirus family, and infection with HIV attenuates the immune system extensively, including in terms of CD4^+^T-cell depletion and diminished immunity. Therefore, HIV-infected patients become susceptible to a wide range of pathogens, and consequently, this leads to high morbidity and mortality [1]. Human pox scabs or dried pus collected from smallpox abscesses was used in the early immunization strategies to prevent smallpox infection from the 10th century onwards in China. Nowadays, as the COVID-19 pandemic has facilitated the development of new vaccines worldwide, vaccination has become the most effective and economical strategy against infectious disease. The representative varieties of inactivated vaccines, attenuated live vaccines, recombinant protein vaccines, toxoid vaccines, polysaccharide vaccines, polysaccharide (protein) combined vaccines, nucleic acid vaccines and viral vector vaccines are shown in Table 1. However, advanced HIV-infected patients are unable to a generate robust immune response to protect against either a natural infection or vaccine antigens [2]. Treatment with antiretroviral therapy (ART) is capable of inhibiting HIV replication, promoting CD4^+^T-cell activation and hence effectively controlling HIV infection, which allows patients to be vaccinated prior to late-stage infection [3]. Therefore, in order to provide better immunization against HIV and improve the quality of life of infected patients, the early timing of administration and vaccine safety and effectiveness warrant concern.

In view of the immunodeficiency of HIV-infected individuals, organizations such as the World Health Organization (WHO), National Institute of Health (NIH), China Center for Disease Control and Prevention (China CDC) and British HIV Association (BHIVA) provide guidelines on the vaccination protocols that account for the potential risks after immunization, as well as disease occurrence and complications, within HIV patients (Table 2) [4,5,6,7,8].

## 2. Inactivated Vaccines

Inactivated vaccines refers to vaccines formulated using pathogenic microorganisms which have been laboratory-cultured and their proliferative ability removed. Currently, the inactivated vaccines on the market for use against various infections have all been proven safe for HIV-infected individuals by international health organizations, including the World Health Organization (WHO), NIH (National Institute of Health), China CDC (China Center for Disease Control and Prevention) and BHIVA (British HIV Association) [4,5,6,7,8]. Simultaneously, patients who receive inactivated vaccinations require further monitoring for undesirable adverse reactions and the provision of instant intervention. Here, we introduce the immunization strategies with the inactivated polio vaccine, the inactivated influenza vaccine and the inactivated COVID-19 vaccine for HIV-infected individuals.

### 2.1. Inactivated Polio Vaccines

The global incidence of paralytic poliomyelitis caused by wild poliovirus has decreased by more than 99% since 1988. However, endemic transmission has still occurred in Pakistan, Afghanistan and Mozambique from 2022 onwards [8,9]. Oral poliovirus vaccines (OPVs) are live virus-based vaccines, and despite their lower toxicity compared to that of the wild-type virus, they can lead to vaccine-derived polioviruses (VDPVs) in patients with dampened immunity [10]. Therefore, the WHO has suggested an anti-poliovirus vaccination program that includes at least one dose of an inactivated polio vaccine (IPV) [8]. In January 2020, China adjusted this strategy into combined immunization, including two doses of an IPV along with two doses of an oral polio vaccine (OPV) [4]. Furthermore, OPVs are prohibited for HIV-infected children in China, regardless of any symptoms indicating impaired immune function. After being vaccinated with three doses of an IPV, the immunodeficient HIV-infected individuals showed a diminished immune response compared to those patients with normal lymph proliferative responses [11]. Another study revealed that 88% of children born to HIV-positive mothers obtained antibodies for three serotypes; all children obtained at least two serotype antibodies after two doses of IPV immunization. No discrepancy in the positive rate or the antibody level was observed between HIV-infected children and non-infected children [12].

### 2.2. Inactivated Influenza Vaccines

Due to their compromised immunity, HIV-infected individuals are more prone to severe or chronic influenza infection and have an increased risk of developing complications compared to healthy individuals [13]; thus, HIV-infected individuals should receive an annual dose of an inactivated influenza vaccine [14]. The trivalent influenza vaccine is the first choice and the most commonly used influenza vaccine for HIV-positive patients [15]. Antiviral therapy is a crucial strategy for inducing an immune response in HIV-positive patients, in which patients with CD4^+^ T cells ≥ 350 cell/mm^3^ are more likely to experience serological changes after being immunized with influenza vaccines compared to patients without therapy [16]. However, a previous study showed that HIV-infected individuals also exhibited diminished immunity in response to regular influenza vaccines when compared to the uninfected population [17]. Data from a meta-analysis show that the administration of two doses of influenza vaccines can induce a more exceptional immune response in HIV patients, with an acceptable safety guarantee [18]. Various strategies have been proposed for compensating for immunodeficiency, for instance, formulating influenza vaccines with adjuvant and booster vaccine doses; however, their effectiveness remains contentious [13].

### 2.3. Inactivated Hepatitis A Vaccines

Hepatitis A virus (HAV) infection is still life-threatening to HIV-infected individuals. Although acute HAV infection is self-limited, the infection that occurs in HIV-infected individuals might lead to a higher risk of mortality from chronic liver disease [19]. Hence, it is recommended that HIV-positive individuals receive a HAV vaccination [20]. According to the China National Health Commission, it has been suggested that HIV-infected children undergo the same HAV immunization procedure as healthy children, in which one dose of an inactivated HAV vaccine (HepA-1) should be administered at 18 months and 24 months after birth, respectively [5]. Children who have not been vaccinated at the recommended age should be immunized with two doses of retroactive HepA-I vaccinations, with an interval no less than 6 months. Adults are also encouraged to receive retroactive vaccination as per the product instructions [21]. A previous study reported that a single dose of an inactivated HAV vaccination in healthy volunteers induced an 80–100% seroconversion rate, and 98–100% of the volunteers produced an HAV-specific immune response after serial vaccination [22]. A variety of studies have shown that the HAV vaccine elicited a lower seroconversion rate and antibody titer in HIV-infected individuals compared to healthy individuals, particularly in volunteers with CD4^+^ T cells < 500/mm^3^ [23,24]. Three administered doses of the HAV vaccine effectively increased the HAV-specific antibody titers in HIV-infected individuals, and no adverse side effects were observed [25,26]. Therefore, the BHIVA advocates a 0–1–6-month, three-dose vaccination schedule for HIV-infected patients who carry CD4^+^ T cells ≤ 350 mm^3^ [27].

### 2.4. Inactivated COVID-19 Vaccines

The global COVID-19 pandemic has resulted in over 770 million infectious cases and more than 7.05 million related deaths (https://covid19.who.int/ accessed on 11 June 2024). The infection and mortality rates of COVID-19 among HIV-infected patients are higher than those in the healthy population [28]. The Joint United Nations Programme on HIV/AIDS (UNAIDS) therefore suggested that HIV-infected patients should be proactively vaccinated against the SARS-CoV-2 virus [29]. Furthermore, the Chinese guidance on SARS-CoV-2 vaccination has also pointed out that both inactivated and recombinant protein vaccines can be safely administered to HIV-infected individuals. Despite HIV-infected patients showing significantly lower IgG seroconversion rates and neutralizing antibody titers compared to those in healthy individuals after the administration of two doses of the inactivated COVID-19 vaccine [30], the immune responses in both groups diminish into a comparable range by 190 days post-vaccination [31].

## 3. Live Attenuated Vaccines

Administering live attenuated vaccines to HIV-infected individuals includes a potential risk. According to the 2021 National Immunization Program of China, the Bacille Calmette–Guérin vaccine (BCG), the live attenuated polio vaccine, the live attenuated encephalitis B vaccine and the live attenuated hepatitis A vaccine cannot be administered to HIV-infected children, regardless of their symptomatic status [4]. On the other hand, live attenuated vaccines are recommended in terms of their good safety for preventing pathogens with high infection rates, depending on the specific conditions. The attenuated vaccines that can be used under certain conditions in HIV infection include mumps vaccines, varicella vaccines, rotavirus vaccines and yellow fever virus vaccines.

### 3.1. A Live Attenuated Measles–Mumps–Rubella Combined Vaccine

The measles–mumps–rubella vaccine (MMR) is the first live attenuated combination vaccine to be developed which contains a mixture of attenuated live measles, mumps and rubella viruses. The 2021 version of the Chinese National Immunization Program allows HIV-infected infants without immunodeficiency to receive two doses of the MMR, according to the procedure for healthy individuals, at 8 months and 18 months, respectively [4]. Meanwhile, immunodeficient infants with AIDS should not be immunized with the MMR. In Zambia, a recent study revealed that administering the MMR to HIV-infected children without ART led to a significantly lower seropositivity (43%) compared to that in healthy children (89%) 27 months after immunization [32].

### 3.2. Live Attenuated Varicella Vaccines

Varicella zoster virus (VZV) is associated with two infectious diseases, chickenpox (the primary infection) and shingles (the secondary infection) [33]. A pilot trial was conducted to evaluate the safety and immunogenicity of a live attenuated varicella vaccine in asymptomatic or mildly symptomatic HIV-infected children. The results showed that the vaccine was well tolerated, with no distinctive local and systemic adverse reaction rates observed compared to those in non-infected children. Following two doses of the vaccine, 60% of HIV-infected children have developed VZV antibodies, and 83% exhibited a VZV-specific cellular immune response (CMI) [34]. A previous study confirmed that the vaccinated population demonstrated milder symptoms when experiencing the breakthrough infection compared to those in unvaccinated individuals [35]. Henceforth, NIH recommends administering the VZV vaccine to HIV-infected children with mild immunodeficiency or without immunodeficiency [21]. Furthermore, NIH advises that previously VZV-vaccinated HIV-infected adults who are over 19 years old should receive two doses of the recombinant herpes zoster vaccine [22].

### 3.3. Live Attenuated Rotavirus Vaccines

Rotavirus is a DNA virus that primarily infects the epithelial cells, is transmitted via fecal–oral routes and that leads to watery diarrhea, fever and vomiting in infants and young children. Severe rotavirus infection can cause dehydration, viral myocarditis and pneumonia and consequently result in fatality [36]. The rotavirus vaccine currently available is formulated with a virulence-attenuated live virus. A retrospective safety study in Kenya demonstrated that there was no increase in the rate of developing a severe adverse reaction with rotavirus immunization in HIV-infected infants compared with that in a placebo group [37]. Accordingly, NIH recommends that asymptomatic HIV-infected children receive live attenuated rotavirus vaccination at ages of 2 months, 3 months and 4 months [21]. Since Africa is an HIV-endemic region, South Africa included the rotavirus vaccine in its national immunization program in August 2009. The diarrhea hospitalization rate in HIV-infected and non-infected children of less than 12 months old significantly dropped from 54.4 to 18.9 (per 1000 population) in the following 5 years [38]. Another study of 76 HIV-positive infants and 126 HIV-exposed (uninfected) infants suggested that the attenuated live rotavirus vaccine was capable of eliciting immune responses in both infected and uninfected infants, and both groups represented similar occurrences of side effects [39].

### 3.4. Live Attenuated Yellow Fever Vaccines

International travelers to epidemic regions are advised to receive one dose of the yellow fever (YF) vaccine at least ten days prior to their departure to reduce the risk of severe and fatal outcomes caused by YF virus. BHIVA has restricted yellow fever vaccination for all HIV-infected persons with CD4^+^T cells < 200/mm^3^ [6]. HIV-positive individuals who travel continuously to or reside in yellow fever epidemic regions should receive a booster every ten years if they fall under the eligible conditions [7].

## 4. Recombinant Protein Vaccines

Recombinant protein vaccines contain artificially synthesized antigens, created through introducing the DNA of the target antigen into engineered cells, where the antigens are synthesized and subsequently extracted for vaccine formulation. All recombinant protein vaccines are suitable for HIV-infected persons across all stages of infection. However, the protectivity of these vaccines depends on the degree of immune impairment of these patients. Therefore, timely vaccination is essential for early-phase HIV-infected people to obtain a high level of protection. Hepatitis B virus (HBV) and human papillomavirus (HPV) vaccines are examples for illustrating these vaccination strategies.

### 4.1. Recombinant Hepatitis B Virus Vaccines

To date, hepatitis B virus infection remains a concerning global health issue, and the vaccination strategies against HBV have been considered some of the most cost-effective public health interventions of their kind [40]. The protectivity offered by recombinant HBV vaccines in healthy adults is able to remain long-lasting at over 90% for 20 years (anti-hepatitis B surface antigen antibodies > 10 mIU/mL), whereas patients who suffer from HIV infection are immunodeficient and incapable of producing prolonged antibodies [41,42]. Consequently, after three doses of 0–1–6-month HBV vaccinations, HIV-infected persons are required to regularly monitor their antibody titers to ensure timely booster vaccinations. Moreover, AIDS patients experiencing severe immunodeficiency should undergo antiretroviral therapy (ART) before an HBV vaccination to enhance their immune response.

### 4.2. Recombinant Human Papillomavirus Vaccines

Human papillomavirus is a common pathogen with over 200 identified types, which have been categorized as low-risk or high-risk based on the viral pathogenesis after infection [43]. Low-risk HPV causes abnormal hyperplasia of the skin and mucosa, such as condyloma, acuminatum and recurrent respiratory papillomatosis. High-risk HPV can lead to various types of cancer, including cervical, oropharyngeal, penile and anal cancer [44,45]. Currently, a total of three types of HPV vaccines (two-, four- and nine-valent) are available on the market. The two-valent HPV vaccine contains the antigens of HPV16 and HPV18 (associated with cervical cancer and anal cancer), and the four-valent HPV vaccine contains the antigens of HPV16, HPV18, HPV6 and HPV11 (responsible for anogenital warts). Nine-valent HPV vaccines are formulated with the additional five types of HPV, HPV31, HPV33, HPV45, HPV52 and HPV58 antigens, which protect against over 90% of HPV-related cervical cancers. Due to the increased risk of HPV-related cervical cancer and anal cancer among HIV-infected individuals, vaccination according to the HPV vaccine instructions is recommended [46]. A tetravalent HPV vaccine provided remarkable protectivity and immunogenicity in 13–27-year-old HIV-positive and -negative adolescents and young adults when they were tested 1 month after three-dose immunization. The seroconversion rate in HIV-infected individuals was 0.85 (95% CI: 0.75–0.95) and 0.91 (0.83–0.99) that of the negative individuals [47]. HIV-infected adolescents who received ART treatment with CD4^+^ T cells > 500 cells/mm^3^ and viral loads < 40 copies/mL attained a 100% seroconversion rate after being immunized with two doses of the nonavalent HPV vaccine [48].

### 4.3. Recombinant Respiratory Syncytial Virus Vaccines

Respiratory syncytial virus (RSV) is a common respiratory tract pathogen that can cause severe infection in infant, elderly or immunodeficient populations. RSV infection does not trigger long-term host immunity and can lead to multiple infections during one’s lifetime. Currently, two types of recombinant RSV prefusion F protein-based vaccines are on the market. The Arexvy vaccine possesses acceptable safety and highly diminishes the risk of RSV-associated lower respiratory tract disease (LRTD) (82.6%) and severe RSV-LRTD (94.1%) [49]. A single-dose vaccination of the RSV prefusion F protein-based vaccine can provide protectivity for two full RSV seasons within a volunteer age group > 60 and showed approximately 68.2% effectivity in decreasing the occurrence of RSV-LRTD and approximately 78.8% effectivity for severe RSV-LRTD [50]. This vaccine has been recommended by NIH as appropriate to apply in HIV-infected individuals aged over 75 and within the high-HIV-risk population aged between 60 and 74 [7]. However, whether the vaccine is safe and effective for HIV-infected individuals is lacking in evidence.

## 5. Toxoid Vaccines

Toxoids are bacterial toxins that have been detoxicated via a chemical or heating procedure while good immunogenicity remains, which can be used in vaccines to induce immunity against pathogenic bacterial components. An example of a toxoid vaccine includes the diphtheria–pertussis–tetanus (DPT) vaccine. Both HIV-positive and -negative infants born to HIV-infected mothers should receive four doses of the DPT vaccine according to the immunization procedure [4]. Bordetella pertussis is a highly transmitted pathogen that leads to pertussis, characterized by whooping cough. A live attenuated pertussis vaccine is unable to provide lifelong protection for vaccinated children. HIV-positive adults infected with B. pertussis will demonstrate a severe course of this disease [51]. The major component of the DPT vaccine that protects against diphtheria is the toxoid vaccine. Immunized HIV-infected individuals given the diphtheria toxoid vaccine demonstrated lower average antibody titers compared to those in the healthy population. The average antibody titers of patients with CD4^+^ T cells < 300 cells/mm^3^ after they received a tetanus toxoid vaccine were less than those in the control group [52].

## 6. Polysaccharide Vaccines

Polysaccharide vaccines are composed of bacterial capsular polysaccharide antigens. The 23-valent pneumococcal polysaccharide vaccine (PPSV23) is safe to be administered at all stages in HIV-infected individuals. However, the immune response elicited by the polysaccharide vaccine in HIV-positive persons is relatively impotent, so NIH recommends that these patients receive a 13-valent pneumococcal polysaccharide conjugate vaccine (PCV13) prior to the administration of the PPSV23 [27]. Children aged 24 to 71 months old who have not completed a full series of PCV13 vaccinations should receive two additional doses of the PCV13, followed by a dose of the PPSV23, administered with at least an eight-week interval. For HIV-infected individuals aged 19 years and older, it is advised to administer one dose of the PCV13 followed by a booster dose of the PPSV23 [53]. In a single-dose protection study of the 13-valent conjugate pneumococcal vaccine in 38 HIV-infected individuals with CD4^+^ T cells > 200 cells/mm^3^, their ELISA results showed that 92.1% of the patients developed a two-fold increase in specific IgG antibody levels 1 month post-vaccination, and 64.7% of the patients were still protected after 12 months [54]. The Food and Drug Administration of the United States has approved the application of the PCV15 and PCV20 in 6-week-old children. The PCV15 induces the same serotype and provides immunogenicity with the same protectivity as the PCV13 in HIV-infected children. Two of the serotypes contained in the PCV15 also showed high immunogenicity. NIH has recommended the PCV15 and PCV20 tetravalent vaccines for use in HIV-infected children. Children who are vaccinated with the PCV15 should be vaccinated with a dose of the PCV20 or PPSV23 8 weeks after their immunization [7].

## 7. Polysaccharide Conjugate Vaccines

Polysaccharide conjugate vaccines consist of covalently bonded polysaccharides and carrier proteins, which enhance the immunogenicity of bacterial polysaccharide antigens. The polysaccharide conjugate vaccines currently available include group A and group C meningococcal conjugate vaccines (PCV), as well as Haemophilus influenzae type B (Hib) conjugate vaccines. According to the guidance from WHO, these vaccines are applicable to individuals infected with HIV [8]. This review will discuss only the immunization strategies for Hib vaccines as an example of the administration of polysaccharide conjugate vaccines in HIV patients.

Invasive Hib disease occurs when Hib infiltrates into the bloodstream, lung parenchyma or meninges. This may lead to clinical manifestations such as septicemia, cellulitis, osteomyelitis and epiglottitis. WHO estimates that Hib disease results in approximately 8.1 million severe cases and 371,000 child deaths annually, in which about 8100 of these children are HIV-positive [55]. In countries with a high prevalence of HIV, the immunization schedules for the Hib vaccine have been modified to up to three doses to enhance antibody titers. Notably, HIV-infected children who commenced ART during infancy (between 6 and 12 weeks) and received three doses of the Hib vaccine, with or without a booster dose, all showed similar Hib antibody titers to those in healthy children in the following two years [56].

## 8. Nucleic Acid Vaccines

mRNA vaccines deliver lipid nanoparticle (NP)-wrapped mRNA into target cells, allowing for the translation of mRNA into antigens, which further elicits a robust pathogen-specific immune response in the host. The first two mRNA vaccines against SARS-CoV-2, BNT162b2 (Pfizer, New York, New York State, USA) and Spikevax (Moderna, Cambridge, MA, USA), demonstrated high efficacy and safety in preventing COVID-19 during clinical trials. Additionally, a COVID-19 mRNA vaccine developed in China was approved through Emergency Use Authorization (EUA) in March 2023. Despite limited research that has evaluated the immune response in HIV patients who were injected with SARS-CoV-2 mRNA vaccines, several studies have demonstrated that HIV-infected individuals with CD4^+^T cells ≥ 200/mm^3^ exhibited lower levels of humoral and cellular immune responses after SARS-CoV-2 mRNA vaccine immunization compared to those in HIV-infected individuals with CD4^+^T cells ≥ 500/mm^3^, while seroconversion was still observed [57,58,59,60]. Furthermore, their administration in severely immunodeficient AIDS patients with a three-dose regimen significantly enhanced the response against SARS-CoV-2 [57].

## 9. Viral Vector Vaccines

Virus vector vaccines use modified, high-safety viral vectors to deliver genetic material encoding pathogen-specific antigens into target cells. Antigens are synthesized directly within the cells and trigger a corresponding immune response. The current viral vector developments are mostly focused on a recombinant adenovirus type 5 (Ad5) vector, a recombinant vesicular stomatitis virus (VSV) vector, a recombinant rabies virus (RABV) vector, a recombinant cytomegalovirus (CMV) vector and a recombinant vaccinia virus (VV) vector. The recombinant Ebola adenovirus vaccine developed in China includes a sequence encoding the Ebola glycoprotein (GP) in the replication-defective vector, which can induce a strong cellular and humoral immune response to prevent Ebola infection [61].

On 7 May 2022, a case of human monkeypox virus (MPV) infection was reported in the United Kingdom. Subsequent cases emerged in Spain, Portugal, the United States and several African countries. The transmission of MVP differs from before, mainly via a human-to-human route, and infectious symptoms include fever, lymphadenopathy, headache and fatigue, along with cutaneous lesions that are commonly observed to arise first in the facial regions, before disseminating throughout the body, including onto the palms and soles [62]. Several cases of atypical MPV infections characterized by oral or anogenital skin lesions were reported during the COVID-19 pandemic and were likely transmitted through sexual contact [63]. One study involving 528 cases of MPV infections at 43 testing sites across 16 countries revealed that approximately 98% of these patients were identified to be homosexual or bisexual men, of whom 41% were HIV-positive [64]. Currently, two types of vaccines are available for preventing MPV infection: replication-deficient modified Vaccinia Ankara (MVA) and the replicative smallpox vaccine (ACAM2000) [64]. ACAM2000 is not suitable for HIV-infected individuals due to its replicative properties; MVA is the preferred vaccine for immunocompromised populations considering its safety guarantee [27].

## 10. Perspectives and Conclusions

In recent decades, significant progress has been made in the technology for vaccines. Ferritin is a natural iron-carrying protein that can self-assemble into a 24-unit sphere. Based on this property, ferritin has been expressed and modified to construct ferritin nanoparticle vaccines. Phase I clinical trial data showed that the SARS-CoV-2 recombinant spike ferritin nanoparticle vaccine adjuvanted with Army Liposome Formulation had good immunogenicity. Ferritin nanoparticles induced neutralizing antibodies against multiple SARS-CoV-2 mutant strains, demonstrating noticeable tolerance and extensive protection [65]. Nipah is a zoonotic disease with a high fatality rate and a high risk of transmission, while Nipah vaccines are still absent on the market. Since the mRNA vaccine technology has been highly improved in recent years, an mRNA Nipah vaccine, mRNA1215, has entered the clinical phase. The mRNA encodes a chimeric antigen which includes a prefusion F glycoprotein and the G glycoprotein of the Malaysia strain of the Nipah virus [66]. An oral recombination *Helicobacter pylori* vaccine fused a urease B subunit and a heat-labile enterotoxin B subunit, showing good immunogenicity and safety in children aged 6–15 years, which could significantly reduce the incidence of H. pylori infections [67]. The application of these new technologies in vaccine research and development may further improve the safety and effectiveness of vaccines and may also provide more options for vaccination in people living with HIV.

This review introduces the recommended vaccines for HIV-infected individuals but lacks a detailed description of certain other vaccines that have been marketed, such as the inactivated hemorrhagic fever with renal syndrome vaccine, the inactivated tick-borne encephalitis live attenuated cholera vaccine, the live attenuated dengue vaccine, the live attenuated typhoid vaccine, the inactivated enterovirus 71 vaccine, the typhoid Vi vaccine and the malaria vaccine. There are no specific vaccination recommendations for these vaccines in populations with HIV.

Immunization serves as a protective strategy for children, adolescents and adults against specific pathogens, significantly reducing the morbidity and mortality due to the corresponding diseases. Due to immunodeficiency, HIV-infected individuals exhibit a limited immune response to vaccinations; consequently, they experience higher morbidity rates, prolonged disease progression, exacerbated symptoms, increased hospitalization rates and elevated mortality due to infections. Therefore, actively promoting vaccination among HIV-positive patients is imperative to decrease the incidence of vaccine-preventable diseases and enhance their quality of life. Given the compromised immune responses observed in populations with HIV, pre-immunization antiretroviral therapy may be administered alongside an increase in the immunogenic dosage to booster immune efficacy; furthermore, their post-vaccination antibody levels should be monitored to facilitate timely immune enhancement interventions.

## Figures and Tables

**Table 1:** Vaccine types and corresponding representative vaccines.

Live Attenuated Vaccine	Inactivated Vaccine	Recombinant Vaccine	Polysaccharide Conjugate Vaccine	Toxoid Vaccine	Viral Vector Vaccine	Polysaccharide Vaccine	Nucleic Acid Vaccine
Live Attenuated Varicella Vaccine	Inactivated Hepatitis A Vaccine	Recombinant Hepatitis B Vaccine	Pneumococcal Polysaccharide Conjugate Vaccine	Tetanus Vaccine	Recombinant Ebola Adenovirus Vaccine	Twenty-Three-Valent Pneumococcal Polysaccharide Vaccine	COVID-19 mRNA Vaccine
Freeze-dried Nasal Spray Live Attenuated Influenza Vaccine	Inactivated Poliovirus Vaccine	Recombinant Hepatitis E Vaccine	Group A and Group C Meningococcal Conjugate Vaccine	Diphtheria, Pertussis and Tetanus Vaccine	Replication-Deficient Modified Vaccinia Ankara	/	/
Live Attenuated Yellow Fever Vaccine	Inactivated COVID-19 Vaccine	Recombinant Human Papillomavirus Vaccine	Haemophilus Influenzae Type B Conjugate Vaccines	/	/	/	/
Oral Poliovirus Vaccine	Rabies Vaccine	Recombinant COVID-19 Vaccine	/	/	/	/	/
Live Attenuated Mumps Vaccine	Inactivated Enterovirus 71 vaccine	Recombinant Herpes Zoster Vaccine	/	/	/	/	/
Live Attenuated Japanese Encephalitis Vaccine	Inactivated Influenza vaccine	Recombinant Respiratory Syncytial VirusVaccine	/	/	/	/	/
Freeze-Dried Live Attenuated Hepatitis a Vaccine	Inactivated Tick-Borne Encephalitis Vaccine	/	/	/	/	/	/
Live Attenuated Rotavirus Vaccine	Inactivated Hemorrhagic Fever with Renal Syndrome Bivalent Vaccine	/	/	/	/	/	/
Live Attenuated Measles–Mumps–Rubella Vaccine	Inactivated Leptospira Vaccine	/	/	/	/	/	/
Bacillus Calmette–Guérin Vaccine	Inactivated Japanese Encephalitis Vaccine	/	/	/	/	/	/

**Table 2:** Recommended vaccines for HIV-infected patients.

Vaccine	WHO	NIH	China CDC	BHIVA
Recombinant Hepatitis B Vaccine	√	√	√	√
Bacillus Calmette–Guérin Vaccine	×	×	×	×
Inactivated Poliovirus Vaccine	√	√	√	√
Oral Poliovirus Vaccine	×	×	×	×
Diphtheria and Tetanus Combined Vaccine	√	√	√	√
Live Attenuated Measles–Mumps–Rubella Vaccine	Prohibition of their use in severely immunocompromised persons	Only in people with CD4^+^T cells > 200/mm^3^	Use only in asymptomatic HIV-infected patients	Only in people with CD4^+^T cells > 200/mm^3^
Inactivated Japanese Encephalitis Vaccine	√	√	√	√
Group A Meningococcal Conjugate Vaccine	√	√	√	√
Group A and Group C Meningococcal Conjugate Vaccine	√	√	√	√
Inactivated Hepatitis A Vaccine	√	√	√	√
Recombinant Human Papillomavirus Vaccine	√	√	/	√
Recombinant Respiratory Syncytial Virus Vaccine	/	>75 years old or 60–74 years old with a high risk	/	/
Inactivated Influenza Vaccine (Split Virion)	√	√	/	√
Live Attenuated Varicella Vaccine	/	Only in people with CD4^+^T cells > 200/mm^3^	/	Only in people with CD4^+^T cells > 200/mm^3^
Hemophilus Influenzae Type B Conjugate Vaccines	√	√	/	√
Pneumococcal Polysaccharide Vaccine	√	√	/	√
Live Attenuated Yellow Fever Vaccine	Prohibition of its use in severely immunocompromised persons	/	/	<60 years old and CD4^+^T cells > 200/mm^3^

√, recommended; ×, not recommended; /, not mentioned; WHO, World Health Organization; NIH, National Institute of Health; China CDC, China Center for Disease Control and Prevention; BHIVA, British HIV Association.
